# Association Between Dipstick Hematuria and Elevated Albuminuria in a Hospital-Based Population with Diverse Chronic Conditions

**DOI:** 10.3390/diagnostics16111678

**Published:** 2026-05-29

**Authors:** Cheewaporn Tanintheerakul, Rungnapa Peerakam, Pharisa Nanthawong, Suwatsin Kittikunnathum, Piyawan Bunpo

**Affiliations:** 1 Clinical Chemistry Laboratory, Diagnostic Service Unit, Maharaj Nakorn Chiang Mai Hospital, Faculty of Medicine, Chiang Mai University, Chiang Mai 50200, Thailand; 2 Division of Clinical Chemistry, Department of Medical Technology, Faculty of Associated Medical Sciences, Chiang Mai University, Chiang Mai 50200, Thailand

**Keywords:** albuminuria, urine albumin-to-creatinine ratio, chronic kidney disease, diabetes mellitus, hypertension, urine dipstick, hematuria

## Abstract

**Background/Objective**: Albuminuria is an early marker of kidney damage and cardiovascular risk. However, data on its prevalence and association with urine dipstick parameters in heterogeneous, hospital-based populations remain limited. The objective of this study was to determine the prevalence of elevated albuminuria measured by the urine albumin-to-creatinine ratio (UACR) in a diverse patient population and evaluate associations with semi-quantitative urine dipstick parameters. **Methods**: This cross-sectional study included 393 adults recruited from a tertiary-care hospital setting, comprising individuals undergoing clinical evaluation and routine health assessments. Given the hospital-based recruitment and enrichment with chronic conditions, the study population represents a high-risk cohort rather than the general population. Spot urine samples were analyzed for the UACR and dipstick parameters. Elevated albuminuria was defined as a UACR ≥ 30 mg/g. Associations were assessed using chi-square tests and multivariate logistic regression, with adjusted odds ratios (aORs) and 95% confidence intervals (CIs). **Results**: Elevated albuminuria was observed in 64% of participants. This high prevalence likely reflects the underlying clinical characteristics of the study population, including a substantial burden of chronic kidney disease and other comorbidities, and should not be interpreted as representative of population-level prevalence. Chronic kidney disease was independently associated with elevated albuminuria (aOR 3.14, 95% CI 1.12–8.86, *p* = 0.030), as was male sex (aOR 1.54, 95% CI 1.01–2.34, *p* = 0.045). Dipstick-positive blood was significantly associated with elevated albuminuria (85% vs. 60% in those without dipstick blood positivity, *p* = 0.005). Dipstick albumin strongly correlated with the UACR (*p* < 0.001), although 22% of individuals with negative dipstick albumin still had an elevated UACR. Other urine dipstick parameters were not significantly associated with elevated albuminuria. **Conclusions**: In this high-risk, hospital-based cohort, elevated albuminuria was common and associated with kidney disease and male sex. Dipstick blood positivity is significantly associated with albuminuria and may warrant further investigation. However, reliance on dipstick testing alone may underestimate disease burden, supporting the need for broader implementation of UACR-based screening strategies.

## 1. Introduction

Albuminuria, defined as abnormal urinary albumin excretion, represents an early and sensitive marker of kidney damage and systemic endothelial dysfunction [1]. The presence of elevated albumin in urine has been established as an independent predictor of progression to chronic kidney disease (CKD) and end-stage renal disease, particularly in high-risk populations such as individuals with diabetes mellitus and hypertension [2,3]. Beyond its renal implications, albuminuria serves as a prognostic indicator for cardiovascular morbidity and mortality, reflecting widespread vascular injury [4].

Current clinical guidelines recommend routine screening for albuminuria in patients with diabetes, hypertension, and other conditions associated with increased CKD risk [5,6]. The urine albumin-to-creatinine ratio (UACR), measured in spot urine samples, has become the preferred method for albuminuria assessment due to its convenience, reproducibility, and strong correlation with 24 h albumin excretion [7]. UACR values are categorized as normoalbuminuria (<30 mg/g creatinine), microalbuminuria (30–299 mg/g), and macroalbuminuria (≥300 mg/g), with each category carrying distinct prognostic implications [8].

Despite guideline recommendations, albuminuria screening remains underutilized in clinical practice [9,10]. Studies from the United States have reported that only 40–60% of eligible patients with diabetes or hypertension receive recommended albuminuria testing [11,12]. This gap between guidelines and practice may contribute to delayed CKD detection and missed opportunities for early intervention with renoprotective therapies.

Reported prevalence of albuminuria varies substantially across populations, reflecting differences in study design, patient characteristics, and testing methodologies. In the general U.S. adult population, approximately 10% demonstrate an elevated UACR, with higher rates observed among individuals with diabetes (31%) and hypertension (18%) [13]. International studies have reported prevalence rates ranging from 5% to 31% depending on the population studied and comorbidity burden [14,15,16].

Semi-quantitative urine dipstick testing offers a rapid, inexpensive screening tool that is widely available in clinical settings. However, the diagnostic accuracy of dipstick proteinuria for detecting albuminuria has been questioned, with studies reporting sensitivities ranging from 30% to 76% and specificities from 80% to 95% [17,18,19]. The relationship between other dipstick parameters, particularly urinary blood (hematuria), and quantitative albuminuria measurements remains incompletely characterized in diverse patient populations.

Understanding the prevalence of albuminuria across different disease categories and its association with routine dipstick parameters can inform screening strategies and improve early detection of kidney disease. This study aimed to: (1) determine the prevalence of elevated albuminuria measured by the UACR in a diverse cohort of patients with various chronic conditions and (2) evaluate associations between quantitative UACR measurements and semi-quantitative urine dipstick findings.

## 2. Materials and Methods

### 2.1. Study Design and Population

This cross-sectional study was conducted at the Clinical Chemistry Laboratory, Diagnostic Service Unit, Maharaj Nakorn Chiang Mai Hospital, Faculty of Medicine, Chiang Mai University, and at the Division of Clinical Chemistry, Department of Medical Technology, Faculty of Associated Medical Sciences, Chiang Mai University. Spot midstream urine samples were obtained from individuals undergoing clinical evaluation and routine health assessments within a tertiary-care hospital setting between November 2025 and February 2026. Due to the hospital-based recruitment strategy, the study population was enriched with patients with chronic medical conditions and therefore represents a high-risk clinical cohort rather than a general screening population. All specimens were anonymized prior to analysis, and no personal identifiers were recorded. No population-based sampling strategy was employed. Therefore, the estimated prevalence of albuminuria in this study reflects the characteristics of a hospital-based cohort and is not intended to represent prevalence in the general population.

The sample size was calculated to detect an association between urine dipstick parameters and albuminuria status using the chi-square test. Based on Cohen’s effect size (w), a two-sided α of 0.05 and 80% power were assumed. For a 2 × 2 contingency table (df = 1), and assuming a small-to-moderate effect size (w = 0.20), the required sample size was estimated to be approximately 393 participants. This estimation was consistent with standard chi-square sample size calculations. Therefore, the inclusion of 393 participants was considered adequate to detect a clinically meaningful association.

The study population consisted of adults aged ≥ 20 years with various chronic medical conditions or those presenting for routine health evaluation. Participants included individuals with diabetes mellitus, kidney disease, essential hypertension, cardiovascular disease, neurological disorders, endocrine disorders, musculoskeletal disorders, dermatologic conditions, systemic lupus erythematosus (SLE), metabolic syndrome, respiratory disorders, infectious diseases, mental health disorders, malignancies, and other conditions, as well as individuals without a specific diagnosis. Kidney disease status was determined based on documented clinical diagnoses in the medical records and was defined independently of the UACR measurements obtained in the present study. The study was conducted in accordance with the Declaration of Helsinki and was approved by the Ethics Research Committee of the Faculty of Medicine, Chiang Mai University (Study code: None-2568-0763).

### 2.2. Data Collection

Demographic information, including age and sex, was collected for all participants. Age was recorded as a continuous variable and subsequently categorized into decade-based groups (20–29, 30–39, 40–49, 50–59, 60–69, 70–79, 80–89, and 90–99 years) for categorical analysis. Primary disease diagnoses were documented based on medical records and clinical assessment. Disease categories with fewer than 10 participants were combined into a composite group for analysis.

### 2.3. Urine Sample Collection and Analysis

Spot urine samples were obtained from all participants for both quantitative UACR measurement and semi-quantitative dipstick analysis. Prior to both analyses, urine specimens were centrifuged at 3000 rpm for 10 min (Kokusan H-36, Saitama, Japan) to remove cellular debris.

#### 2.3.1. Quantitative Albuminuria Assessment

Quantitative analyses were performed using the Roche Cobas^®^ c503 analyzer (Roche Diagnostics, Mannheim, Germany). Urinary albumin was measured by an immunoturbidimetric assay (Tina-quant^®^ Albumin Gen.2, Roche Diagnostics, Mannheim, Germany), and urinary creatinine was determined using an enzymatic method (Creatinine Plus ver.2, Roche Diagnostics, Mannheim, Germany). Calibration and internal quality control procedures were performed according to the manufacturer’s recommendations. The urinary creatinine assay was traceable to isotope dilution mass spectrometry (IDMS)-aligned reference methods, while the urinary albumin assay utilized manufacturer-provided calibration materials designed for standardized quantitative urinary albumin measurement. Participants were categorized according to UACR values as follows: normoalbuminuria (<30 mg/g), microalbuminuria (30–299 mg/g), and macroalbuminuria (≥300 mg/g). For analytical purposes, microalbuminuria and macroalbuminuria were combined into a single “elevated albuminuria” category to compare against normoalbuminuria.

#### 2.3.2. Semi-Quantitative Urine Dipstick Analysis

Semi-quantitative urinalysis was performed using the Dirui H13-Cr strip (Dirui Industrial Co., Ltd., Changchun, China) according to the manufacturer’s instructions. Urinary parameters were recorded according to the manufacturer’s grading scale, including blood (negative, trace, approximately 10–200 erythrocytes/µL), albumin (≤10 to ≥150 mg/L), glucose (negative to 56 mmol/L), ketones (negative to 7.8 mmol/L), leukocytes (negative to approximately 500 cells/µL), nitrite (negative/positive), bilirubin (negative to ≥51 µmol/L), urobilinogen (<3.4 to ≥17.0 µmol/L), creatinine (0.9 to >26.5 mmol/L), specific gravity (1.000–1.030), and pH (5.0–8.5).

### 2.4. Statistical Analysis

Descriptive statistics were calculated for all study variables. Continuous variables, including age and the UACR, were reported as median (range) and mean ± standard deviation (SD), respectively, while categorical variables, including sex, underlying conditions, and urine dipstick parameters, were presented as frequencies and percentages. The normality of continuous data was evaluated using the Shapiro–Wilk test and visual inspection of data distribution. As the data were not normally distributed, the Mann–Whitney U test was used to compare continuous variables between groups. Categorical variables were compared using the chi-square test.

To identify independent factors associated with elevated albuminuria, multivariate logistic regression analysis was performed. Variables were selected for inclusion in the multivariable logistic regression model based on clinical relevance and/or a *p*-value < 0.10 in univariate analysis to avoid excluding potentially important predictors. The final model included age, sex, diabetes mellitus, hypertension, kidney disease, and cardiovascular disease. No missing data were identified for the variables included in the regression analysis; therefore, all participants were included in the final multivariable model. Prior to model fitting, multicollinearity among independent variables was assessed using variance inflation factors (VIFs) and correlation analysis. No evidence of significant multicollinearity was observed, and all selected variables were retained in the final model. Adjusted odds ratios (aORs) with 95% confidence intervals (CIs) were calculated. A *p*-value < 0.05 was considered statistically significant. All statistical analyses were performed using IBM SPSS Statistics version 17.0 (IBM Corp., Armonk, NY, USA).

## 3. Results

### 3.1. Baseline Characteristics of Participants

A total of 393 participants were included in the analysis, of whom 250 (64%) had elevated albuminuria. This relatively high prevalence reflects the hospital-based nature of the cohort, which included individuals with multiple chronic conditions and is therefore not representative of the general population. The median age did not differ significantly between the normoalbuminuria and elevated albuminuria groups (64 vs. 64 years, Mann–Whitney U test, *p* = 0.397). The proportion of male participants was higher in the elevated albuminuria group than in the normoalbuminuria group (68% vs. 32%), although this difference did not reach statistical significance (χ^2^ test, *p* = 0.069).

Regarding underlying conditions, kidney disease was markedly more frequent among participants with elevated albuminuria (85% vs. 15%; *p* = 0.017), whereas cardiovascular disease was slightly more common in the normoalbuminuria group (51% vs. 49%; *p* = 0.039). Although diabetes mellitus and hypertension were more frequently observed among participants with elevated albuminuria compared with those with normoalbuminuria, neither association reached statistical significance (diabetes mellitus: *p* = 0.484; hypertension: *p* = 0.610). Participants without a documented diagnosis and those categorized under “other conditions” showed comparable distributions between groups. The “other conditions” category included a heterogeneous group of disorders aggregated to improve statistical stability (Table 1).

### 3.2. Distribution of Albuminuria Categories

Based on the urinary albumin-to-creatinine ratio (UACR), 143 participants (36%) were classified as normoalbuminuria, 177 (45%) as microalbuminuria, and 73 (19%) as macroalbuminuria. The mean UACR values increased progressively across categories, from 9 ± 7.5 mg/g in normoalbuminuria to 119 ± 76.5 mg/g in microalbuminuria and 1239 ± 1143.8 mg/g in macroalbuminuria, reflecting a wide dispersion in the higher range (Table 2).

### 3.3. Factors Associated with Elevated Albuminuria

Multivariate logistic regression analysis identified male sex and kidney disease as independent factors associated with elevated albuminuria (Table 3). Male participants had a 54% higher odds of elevated albuminuria compared to females (adjusted OR 1.54; 95% CI 1.01–2.34; *p* = 0.045). Although sex was not significant in univariate analysis (Table 1), it became significant after multivariate adjustment, suggesting confounding effects. Kidney disease showed a strong association, with more than a threefold increased odds (adjusted OR 3.14; 95% CI 1.12–8.86; *p* = 0.030). In contrast, age, diabetes mellitus, and hypertension were not significantly associated with elevated albuminuria in the adjusted model. Notably, cardiovascular disease, which demonstrated a statistically significant inverse association with albuminuria in the univariate analysis (Table 1), was no longer significant in the multivariate model (adjusted OR 0.51, 95% CI 0.25–1.03, *p* = 0.061). Although the direction of association remained inverse, the attenuation of statistical significance suggests that the crude association observed in Table 1 may have been influenced by confounding factors.

### 3.4. Association Between Urine Dipstick Parameters and Albuminuria

Significant associations were observed between certain dipstick parameters and elevated albuminuria. Participants with dipstick-positive blood (trace or higher) had a higher prevalence of elevated albuminuria compared to those with negative results (85% vs. 60%, *p* = 0.005). Dipstick albumin showed a strong correlation with UACR-defined albuminuria. Nearly all participants with dipstick albumin ≥150 mg/L had elevated albuminuria (99%), compared to only 22% among those with ≤10 mg/L (*p* < 0.001). Urine glucose was not significantly associated with albuminuria status (*p* = 0.111) (Table 4).

### 3.5. Other Urine Parameters

No significant associations were found between elevated albuminuria and other urine dipstick parameters, including ketones, leukocytes, nitrite, bilirubin, urobilinogen, creatinine, specific gravity, and pH (all *p* > 0.05) (Table 5 and Supplementary Tables S1–S10).

## 4. Discussion

This cross-sectional study of 393 participants with diverse chronic conditions demonstrated substantial variation in albuminuria prevalence across disease categories and a significant association with dipstick blood positivity. These findings highlight the clinical relevance of albuminuria screening and its potential role in early detection of chronic kidney disease (CKD) among high-risk populations.

### 4.1. Albuminuria Prevalence Across Clinical Conditions

The observed prevalence of elevated albuminuria (64%) in this study is substantially higher than estimates reported in population-based studies. This discrepancy is primarily attributable to the hospital-based design and the inclusion of a high-risk population enriched with chronic medical conditions, particularly chronic kidney disease. As such, the prevalence reported here should be interpreted within the context of a clinical cohort and not extrapolated to the general population. Among participants with diabetes mellitus, 68% exhibited elevated albuminuria, which is considerably higher than the 31% prevalence reported in a recent NHANES analysis of U.S. adults with diabetes [13]. Likewise, the prevalence among individuals with hypertension in our cohort was 62%, compared with 18% in the NHANES hypertensive population [13]. These differences likely reflect variations in clinical characteristics between a hospital-based population and a community-based survey, including disease duration, severity, comorbidities, and treatment status. In participants with established kidney disease, the prevalence of elevated albuminuria reached 85%, consistent with the well-recognized role of albuminuria as both a marker and mediator of CKD progression [20,21]. Albuminuria is widely accepted as an early manifestation of nephropathy and an independent predictor of progression to end-stage renal disease [3,22], supporting its continued use in CKD risk stratification and monitoring.

Interestingly, 60% of participants without a documented diagnosis also demonstrated elevated albuminuria. This finding may indicate the presence of subclinical kidney impairment or undiagnosed conditions such as diabetes or hypertension. It underscores the potential value of opportunistic albuminuria screening, even among individuals not previously identified as high risk, particularly those with predisposing factors such as advanced age, obesity, or a family history of kidney disease [23,24,25].

In this study, CKD emerged as a strong independent predictor of albuminuria, while diabetes mellitus, despite being a well-established risk factor, was not significantly associated after multivariate adjustment. This finding warrants careful interpretation in the context of underlying pathophysiology and model structure. First, the strong association between CKD and albuminuria is biologically plausible. Albuminuria reflects glomerular damage and increased permeability of the filtration barrier, which are hallmark features of CKD. As such, CKD represents a more proximal pathological state directly linked to albumin leakage, whereas DM is a more distal risk factor that contributes to kidney damage over time through metabolic and hemodynamic mechanisms. Second, the lack of statistical significance for DM may be explained by collinearity and mediation effects. Diabetes is a leading cause of CKD, and the inclusion of CKD in the multivariate model may have attenuated the independent effect of DM. In this context, CKD may act as an intermediate variable in the causal pathway between DM and albuminuria, resulting in a phenomenon of over-adjustment. Third, heterogeneity within the diabetic population may have further diluted the observed association. The study did not account for important clinical parameters such as duration of diabetes, glycemic control (e.g., HbA1c), or treatment regimens, all of which are known to influence the risk of albuminuria. Consequently, the binary classification of DM may not adequately capture disease severity or progression.

The relatively weak association between CVD and albuminuria should also be interpreted cautiously. Possible explanations include residual confounding, limited subgroup size, heterogeneity of cardiovascular conditions, and the hospital-based nature of the study population. In addition, patients with established CVD may have been receiving renoprotective therapies such as angiotensin-converting enzyme inhibitors (ACE inhibitors), angiotensin receptor blockers (ARBs), or sodium-glucose cotransporter-2 (SGLT2) inhibitors, which could reduce urinary albumin excretion and attenuate the observed association. Similarly, the absence of significant associations with age and hypertension may reflect residual confounding, limited statistical power, or unmeasured clinical variables.

Importantly, the relatively low explanatory power of the model indicates that other factors, such as obesity, smoking status, inflammatory markers, or renal function parameters (e.g., estimated glomerular filtration rate), may play a significant role in the development of albuminuria and should be considered in future studies.

### 4.2. Dipstick Blood Positivity and Albuminuria: A Significant Association

One of the key findings of this study is the statistically significant association between dipstick-detected urinary blood and elevated albuminuria. Among participants with negative dipstick blood results, 60% had elevated albuminuria, whereas 85% of those with trace or higher dipstick-positive blood demonstrated elevated albuminuria. This association has not been consistently reported in the albuminuria screening literature reviewed.

The biological basis for this association may involve several mechanisms. Dipstick-positive blood and albuminuria may occur as a consequence of glomerular injury, which increases permeability of the renal filtration barrier to both red blood cells and albumin [26]. Conditions such as glomerulonephritis, diabetic nephropathy with glomerular hyperfiltration injury, and hypertensive nephrosclerosis can manifest with both findings [27]. Additionally, endothelial dysfunction, a common pathway in diabetes, hypertension, and cardiovascular disease, may contribute to both increased glomerular permeability to albumin and microhemorrhage [28]. However, dipstick-positive blood is nonspecific and may also arise from non-glomerular causes; therefore, the observed association should be interpreted cautiously.

From a clinical screening perspective, this association suggests that dipstick-detected hematuria may serve as a readily available indicator that warrants quantitative UACR measurement. While dipstick proteinuria has been studied extensively as a screening tool for albuminuria, with reported sensitivities of 30–76% [17,18,19], the potential utility of dipstick blood positivity as a screening indicator has received less attention. Our findings suggest that the presence of dipstick-positive blood on routine urinalysis should prompt clinicians to obtain quantitative albuminuria assessment, even in the absence of dipstick proteinuria.

However, it is important to note that the supplied literature did not provide consistent evidence on the hematuria–albuminuria relationship, and our finding requires validation in independent cohorts. The association may be influenced by the specific disease mix in our population and may not generalize to all clinical settings.

### 4.3. Dipstick Albumin Performance

As expected, semi-quantitative dipstick albumin showed a highly significant association with the quantitative UACR. However, the performance characteristics reveal important limitations. Among participants with dipstick albumin ≤10 mg/L, 22% still had elevated albuminuria by the UACR, indicating that dipstick testing misses a substantial proportion of cases with microalbuminuria. This finding is consistent with previous studies demonstrating that dipstick proteinuria has limited sensitivity for detecting microalbuminuria [17,29,30]. In addition, the diagnostic performance of urine dipstick testing may vary considerably among different manufacturers due to differences in reagent composition, analytical sensitivity, color interpretation systems, calibration methods, and threshold definitions for positivity. Previous studies have reported variability in sensitivity and specificity across commercially available dipstick assays, which may affect comparability between studies and influence screening accuracy in clinical practice [18,19]. Therefore, caution is warranted when interpreting dipstick-based albuminuria results across different testing platforms, particularly in low-level albuminuria detection.

In a Japanese cohort study, using at least one “trace” result on three dipstick measurements yielded sensitivity of 0.56 and specificity of 0.80 for microalbuminuria detection [21]. A single urine protein-to-creatinine ratio (UPCR) measurement outperformed the three-dipstick strategy with sensitivity of 0.76 and specificity of 0.84 [21]. These findings support the conclusion that quantitative measurements (UACR or UPCR) are superior to dipstick testing for albuminuria screening, particularly for detecting microalbuminuria in its early stages when intervention may be most effective [31,32].

The clinical implication is that dipstick testing alone is insufficient for comprehensive albuminuria screening in high-risk populations. While dipstick albumin ≥150 mg/L showed 99% concordance with an elevated UACR in our study, relying on dipstick positivity to triage patients for UACR testing, a strategy employed in some large clinic studies [3], will underestimate true prevalence and miss opportunities for early intervention.

Although urine dipstick albumin and dipstick-positive blood were significantly associated with elevated albuminuria, these findings should be interpreted cautiously because formal diagnostic accuracy analyses were not performed. The results suggest that these dipstick parameters may serve as practical indicators for further quantitative UACR assessment in routine clinical practice. However, dipstick-positive blood does not necessarily confirm true hematuria, as the test detects the peroxidase activity of hemoglobin and myoglobin rather than erythrocytes directly. Future studies should include confirmatory microscopic urinalysis and diagnostic performance analyses, including sensitivity, specificity, predictive values, and receiver operating characteristic (ROC)/area under the curve (AUC) analysis, to better define the clinical utility of these dipstick parameters.

### 4.4. Lack of Association with Other Dipstick Parameters

Contrary to expectations, we found no significant associations between albuminuria and several dipstick parameters that might be hypothesized to correlate with kidney disease or metabolic dysfunction. Urinary glucose showed no significant association, despite the strong link between diabetes and albuminuria. This likely reflects that glycosuria occurs only when blood glucose exceeds the renal threshold (typically ~180 mg/dL), whereas albuminuria can develop at any level of glycemic control in diabetic patients and is more closely related to duration of diabetes, blood pressure control, and individual susceptibility to diabetic nephropathy [33,34].

Similarly, urinary leukocytes, nitrite, ketones, and other parameters showed no significant associations with albuminuria status. These findings suggest that routine dipstick parameters beyond albumin and blood provide limited additional information for predicting quantitative albuminuria. This has practical implications for screening protocols: a focused approach measuring the UACR directly, with attention to dipstick blood positivity when present, may be more efficient than attempting to use multiple dipstick parameters as indirect indicators.

### 4.5. Demographic Factors

Interestingly, sex was not significantly associated with albuminuria in univariate analysis but became significant after multivariate adjustment. This suggests the presence of confounding effects from other variables, particularly chronic kidney disease and cardiovascular comorbidities, which may differ by sex. Adjustment for these factors likely unmasked the independent association between male sex and albuminuria. Previous population studies have reported mixed findings regarding sex differences in albuminuria prevalence, with some showing higher rates in males and others finding no difference after adjusting for comorbidities [35,36]. Therefore, the observed association should be interpreted with caution due to the observational nature of the study and the potential for residual confounding.

In contrast, age showed no significant association in either univariate or multivariate analyses. This finding may reflect the relatively homogeneous age distribution of the study population, with the majority of participants clustered in older age groups, thereby limiting the ability to detect age-related differences. Additionally, the effect of age on albuminuria may be mediated through comorbid conditions such as hypertension, diabetes, and CKD, which were included in the multivariate model.

Taken together, these findings suggest that while age alone may not independently predict albuminuria in this cohort, sex-specific differences and comorbidity burden play a more important role in determining risk.

### 4.6. Clinical and Public Health Implications

This was a hospital-based convenience sample enriched with chronic conditions and is not representative of the general population. The high prevalence of elevated albuminuria in this diverse chronic conditions underscores the importance of systematic screening in high-risk groups. Current guidelines recommend annual albuminuria testing for patients with diabetes and hypertension [5,6], yet screening rates remain suboptimal, with only 40–60% of eligible patients receiving recommended testing [11,12]. Our findings reinforce the need for improved implementation of guideline-recommended screening.

The association between dipstick blood positivity and albuminuria suggests a potential two-step screening approach: routine dipstick testing (which is inexpensive and widely available) could identify patients with hematuria or dipstick proteinuria who should then receive quantitative UACR measurement. This approach might improve screening efficiency in resource-limited settings, though it would still miss some cases of isolated microalbuminuria without dipstick-positive blood.

For patients found to have elevated albuminuria, evidence-based interventions are available. ACE inhibitors and ARBs have demonstrated efficacy in reducing albuminuria and slowing CKD progression in diabetic and non-diabetic kidney disease [37,38]. SGLT2 inhibitors have emerged as additional renoprotective agents with benefits in reducing albuminuria and CKD progression [39]. Early detection through systematic screening enables timely initiation of these therapies.

### 4.7. Limitations

This study has several important limitations. First, the hospital-based design introduces selection bias, as the study population was enriched with individuals with chronic medical conditions, particularly chronic kidney disease. Consequently, the observed prevalence of albuminuria is likely overestimated and not generalizable to the broader population. Second, albuminuria was assessed using a single spot urine sample without repeat confirmation, which may have led to misclassification due to transient albuminuria. Furthermore, the present study was not specifically designed to assess the diagnostic performance of urine dipstick protein or dipstick-positive blood; therefore, detailed analyses of sensitivity, specificity, predictive values, and ROC/AUC were not conducted. In addition, dipstick-positive blood findings were not confirmed by microscopic examination for urinary red blood cells, limiting differentiation between true hematuria and the presence of free hemoglobin or myoglobin. Accordingly, the results should be interpreted as representing dipstick blood positivity rather than confirmed hematuria. Third, several clinically relevant variables, including estimated glomerular filtration rate (eGFR), diabetes duration, HbA1c, body mass index (BMI), medication use, urinary tract infection status, menstruation status, and treatment history, were unavailable in this study. The absence of these variables limited comprehensive risk adjustment and may have resulted in residual confounding. Finally, limited published evidence on the hematuria–albuminuria relationship constrained contextual interpretation of this finding.

## 5. Conclusions

In conclusion, elevated albuminuria was associated with male sex and pre-existing kidney disease in this hospital-based cohort, while urine dipstick albumin and dipstick-positive blood showed significant associations with UACR-defined albuminuria. These findings suggest that routine dipstick parameters may provide useful preliminary information for identifying individuals who could benefit from further quantitative UACR assessment, particularly in resource-limited settings. However, given the cross-sectional design, hospital-based recruitment, and use of a single spot urine measurement, the findings should be considered hypothesis-generating and require validation in larger, prospective, and more representative populations.

## Figures and Tables

**Table 1:** Baseline characteristics of participants according to albuminuria status (*n* = 393).

Characteristic	Normoalbuminuria (*n* = 143)	Elevated Albuminuria (*n* = 250)	*p*-Value *
Age, median (age range, years)	64 (31–88)	64 (20–92)	0.397
Sex			
Male, *n* (%)	66 (32%)	143 (68%)	0.069
Female, *n* (%)	77 (42%)	107 (58%)	
Underlying conditions, *n* (%)			
Diabetes mellitus	31 (32%)	67 (68%)	0.484
Hypertension	37 (38%)	60 (62%)	0.610
Kidney disease	8 (15%)	46 (85%)	0.017
Cardiovascular disease	21 (51%)	20 (49%)	0.039
No diagnosis	19 (40%)	28 (60%)	–
Other conditions †	27 (48%)	29 (52%)	–

Data are presented as *n* (%) unless otherwise indicated. * Continuous variables were compared using the Mann–Whitney U test. Categorical variables were compared using the chi-square test. † Other conditions include obesity, dyslipidemia, neurological, endocrine, musculoskeletal, dermatologic, autoimmune (including SLE), infectious, respiratory, psychiatric disorders, malignancies, and miscellaneous conditions with small sample sizes (*n* < 10 per category), which were combined to improve statistical stability and interpretability.

**Table 2:** Distribution of albuminuria categories based on UACR (*n* = 393).

Category	UACR (mg/g)	Mean ± SD	*n* (%)
Normoalbuminuria	<30	9 ± 7.5	143 (36%)
Microalbuminuria	30–299	119 ± 76.5	177 (45%)
Macroalbuminuria	≥300	1239 ± 1143.8	73 (19%)

Data are presented as Mean ± SD.

**Table 3:** Multivariate logistic regression analysis of factors associated with elevated albuminuria.

Variable	Adjusted OR	95% CI	*p*-Value
Age (per year)	1.00	0.98–1.02	0.963
Sex (male vs. female)	1.54	1.01–2.34	0.045
Diabetes mellitus	1.22	0.70–2.13	0.489
Hypertension	0.93	0.54–1.60	0.785
Kidney disease	3.14	1.12–8.86	0.030
Cardiovascular disease	0.51	0.25–1.03	0.061

OR = odds ratio; CI = confidence interval, variables included in the model: age, sex, diabetes mellitus, hypertension, CKD, and cardiovascular disease, statistical significance defined as *p* < 0.05.

**Table 4:** Association between selected urine dipstick parameters and albuminuria.

Parameter	Category	Normoalbuminuria *n* (%)	Elevated Albuminuria *n* (%)	*p*-Value
Blood	Negative	134 (40%)	198 (60%)	0.005
	Trace or higher	9 (15%)	52 (85%)	
Albumin	≤10 mg/L	63 (78%)	18 (22%)	<0.001
	11–29	12 (100%)	0 (0%)	
	30	27 (47%)	30 (53%)	
	31–79	25 (61%)	16 (39%)	
	80	4 (13%)	27 (87%)	
	81–149	10 (25%)	30 (75%)	
	≥150 mg/L	2 (1%)	129 (99%)	
Glucose	Negative	110 (40%)	165 (60%)	0.111
	≥5.6 mmol/L	33 (28%)	85 (72%)	

Categorical variables were compared using the chi-square test, statistical significance defined as *p* < 0.05.

**Table 5:** Association between other urine dipstick parameters and albuminuria.

Parameter	*p*-Value
Ketones	0.426
Leukocytes	0.514
Nitrite	0.582
Bilirubin	0.287
Urobilinogen	0.434
Creatinine	0.589
Specific gravity	0.094
pH	0.170

Categorical variables were compared using the chi-square test, statistical significance defined as *p* < 0.05.

## Data Availability

The original contributions presented in this study are included in the article/Supplementary Materials. Further inquiries can be directed to the corresponding author.

## Supplementary Materials

The following supporting information can be downloaded at: https://www.mdpi.com/article/10.3390/diagnostics16111678/s1, Table S1. Association between urinary blood and albuminuria; Table S2. Association between dipstick albumin and UACR-defined albuminuria; Table S3. Association between urinary glucose and albuminuria; Table S4. Association between urobilinogen and albuminuria; Table S5. Association between bilirubin and albuminuria; Table S6. Association between ketones and albuminuria; Table S7. Association between urinary creatinine and albuminuria; Table S8. Association between nitrite and albuminuria; Table S9. Association between leukocytes and albuminuria; Table S10. Association between specific gravity, pH, and albuminuria.
