# Supplementary material for: Association Between Dipstick Hematuria and Elevated Albuminuria in a Hospital-Based Population with Diverse Chronic Conditions
Source: Diagnostics (Basel). 2026 May 29;16(11):1678. doi: 10.3390/diagnostics16111678 (PMC13257344; doi:10.3390/diagnostics16111678)
Supplement: Supplementary file 1 [file diagnostics-16-01678-s001.zip › diagnostics-4306783-supplementary.pdf]

Supplementary Tables S1-S10

Supplementary Table S1. Association between urinary blood and albuminuria

Blood (RBC/μL)	Normoalbuminuria n (%)	Elevated albuminuria n (%)	Total
Negative	134 (40.4)	198 (59.6)	332
Trace	1 (5.0)	19 (95.0)	20
~10	0 (0.0)	1 (100.0)	1
~25	3 (18.7)	13 (81.3)	16
~80	2 (15.4)	11 (84.6)	13
~200	3 (27.3)	8 (72.7)	11

Data are presented as n (%). $p = 0.005$ (chi-square test).

Supplementary Table S2. Association between dipstick albumin and UACR-defined albuminuria

Dipstick albumin (mg/L)	Normoalbuminuria n (%)	Elevated albuminuria n (%)	Total
≤10 mg/L	63 (77.8)	18 (22.2)	81
11-29	12 (100.0)	0 (0.0)	12
30	27 (47.4)	30 (52.6)	57
31-79	25 (61.0)	16 (39.0)	41
80	4 (12.9)	27 (87.1)	31
81-149	10 (25.0)	30 (75.0)	40
≥150 mg/L	2 (1.5)	129 (98.5)	131

Data are presented as n (%). $p < 0.001$ (chi-square test).

Supplementary Table S3. Association between urinary glucose and albuminuria

Glucose (mmol/L)	Normoalbuminuria n (%)	Elevated albuminuria n (%)	Total
Negative	110 (40.0)	165 (60.0)	275
5.6	0 (0.0)	3 (100.0)	3
14	2 (20.0)	8 (80.0)	10
28	18 (25.4)	53 (74.6)	71
29–55	0 (0.0)	1 (100.0)	1
56	13 (39.4)	20 (60.6)	33

Data are presented as n (%). $p = 0.111$ (chi-square test).

Supplementary Table S4. Association between urobilinogen and albuminuria

Urobilinogen (μmol/L)	Normoalbuminuria n (%)	Elevated albuminuria n (%)	Total
<3.4	36 (34.3)	69 (65.7)	105
3.4	36 (43.4)	47 (56.6)	83
3.5–16.9	65 (35.9)	116 (64.1)	181
≥17.0	6 (25.0)	18 (75.0)	24

Data are presented as n (%). $p = 0.434$ (chi-square test).

Supplementary Table S5. Association between bilirubin and albuminuria

Bilirubin (μmol/L)	Normoalbuminuria n (%)	Elevated albuminuria n (%)	Total
Negative	17 (33.3)	34 (66.7)	51
<17	49 (35.8)	88 (64.2)	137
17	73 (36.4)	127 (63.5)	200
18–50	2 (66.7)	1 (33.3)	3
≥51	2 (100.0)	0 (0.0)	2

Data are presented as n (%). p = 0.287 (chi-square test).

Supplementary Table S6. Association between ketones and albuminuria

Ketones (mmol/L)	Normoalbuminuria n (%)	Elevated albuminuria n (%)	Total
Negative	137 (36.3)	240 (63.7)	377
0.5	4 (31.8)	9 (69.2)	13
3.9	1 (50.0)	1 (50.0)	2
7.8	1 (100.0)	0 (0.0)	1

Data are presented as n (%). p = 0.426 (chi-square test).

Supplementary Table S7. Association between urinary creatinine and albuminuria

Creatinine (mmol/L)	Normoalbuminuria n (%)	Elevated albuminuria n (%)	Total
≤4.4	34 (35.8)	61 (64.2)	95
4.5–17.6	22 (28.2)	56 (71.8)	78
≥17.7	87 (39.5)	133 (60.5)	220

Data are presented as n (%). p = 0.589 (chi-square test).

Supplementary Table S8. Association between nitrite and albuminuria

Nitrite	Normoalbuminuria n (%)	Elevated albuminuria n (%)	Total
Negative	134 (36.8)	230 (63.2)	364
Positive	9 (31.0)	20 (69.0)	29

Data are presented as n (%). p = 0.582 (chi-square test).

Supplementary Table S9. Association between leukocytes and albuminuria

Leukocytes (cells/µL)	Normoalbuminuria n (%)	Elevated albuminuria n (%)	Total
Negative	107 (34.9)	200 (65.1)	307
~15	16 (50.0)	16 (50.0)	32
~70	13 (43.3)	17 (56.7)	30
~125	7 (30.4)	16 (69.6)	23
~500	0 (0.0)	1 (100.0)	1

Data are presented as n (%). p = 0.514 (chi-square test).

Supplementary Table S10. Association between specific gravity, pH, and albuminuria

Parameter	Category	Elevated albuminuria n (%)	p-value
Specific gravity	1.000–1.010	53–60%	0.094
Specific gravity	1.015–1.030	62–70%	
pH	5.0–6.0	65–68%	0.170
pH	≥6.5	50–82%	

Data are summarized as percentages. Chi-square test used for comparisons.
